# Mean platelet volume/platelet count ratio predicts severe pneumonia of COVID‐19

**DOI:** 10.1002/jcla.23607

**Published:** 2020-10-31

**Authors:** Qingyang Zhong, Jie Peng

**Affiliations:** ^1^ Department of Infectious Diseases Nanfang Hospital Southern Medical University Guangzhou China

**Keywords:** COVID‐19, inflammation, novel coronavirus pneumonia, platelet mean volume/platelet count ratio, severe pneumonia

## Abstract

**Background:**

Although platelet mean volume/platelet count ratio (MPR) is considered to be a crucial marker of inflammatory and infectious diseases, the relationship between MPR and novel coronavirus infectious disease 2019 (COVID‐19) remains unclear.

**Methods:**

In this retrospective study, 85 patients with confirmed COVID‐19 were enrolled and divided into low and high MPR group. Data from repeated measures were compared by the generalized estimating equations. Cox regression analyses were performed to assess the impact of MPR on the incidence of severe pneumonia (SP), with inverse probability of treatment weighting (IPTW) used to reduce confounding bias. The primary outcome is the incidence of SP of COVID‐19.

**Results:**

During follow‐up, 17 (20.0%) patients were developed to SP. Compared with mild patients, patients with SP developed showed a higher MPR level at baseline, day 1, day 2, and day 3 after admission (*P* = .005, *P* = .015, *P* = .009, and *P* = .032, respectively). Kaplan‐Meier method showed a higher incidence of SP in the high MPR group than the low MPR group (log‐rank test = 10.66, *P* = .001). After adjustment, high MPR was associated with an elevated incidence of SP (HR, 5.841, 95% CI, 1.566‐21.791, *P* = .009). The IPTW method also suggested that MPR was a significant factor related to the incidence of SP (HR, 8.337, 95% CI, 4.045‐17.182, *P* < .001).

**Conclusion:**

High MPR level is an independent risk factor for severe pneumonia in patients with COVID‐19.

## Graphical abstract

High MPR level is an independent risk factor for disease progression in patients with COVID‐19. Patients with SP developed tended to have higher MPR levels than mild patients in the early stage after admission.

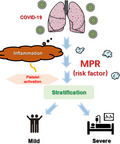

## 1 INTRODUCTION

From December 2019, a virus, named novel coronavirus (2019‐nCOV), attracting the attention of the whole world.^1^ As on June 28, 2020, 9 825 539 cases were reported across the globe, with deaths up to 495 388. COVID‐19, a zoonosis virus closely related to bat SARS‐like coronavirus strain, is mainly transmitted through respiratory droplets and contact, while the oral route of feces remains to be determined.^2^, ^3^ Common symptoms include fever, dry cough/expectoration, fatigue, upper respiratory congestion, ecphysesis, gastrointestinal symptoms, and myalgia/arthralgia.^4^, ^5^ Most patients present mild symptoms, but it may progress to severe symptoms in some patients (especially the elderly and/or patients with comorbidity).^4^ Severe patients develop rapidly into acute respiratory failure, acute respiratory distress syndrome (ARDS), septic shock, metabolic acidosis, and coagulation disorders.^6^ The prognosis of patients with severe pneumonia (SP) of COVID‐19 is worse than that of patients with mild type. Early recognization of the risk factors of SP contributes to antecedent intervention, which shows crucial clinical significance for the treatment and prognosis of patients. SP associates with plenty of abnormal laboratory tests, which indicate different injuries of specific organs.^7^ Complex pathophysiology involves infection and systemic inflammatory response, causing a series of reactions such as coagulation activation, liver damage, myocardial damage, and kidney damage.^8^ Platelets play a significant role in the procedure of inflammation and coagulation, activated platelets release a great number of substances, which belong to the key factors of inflammation.^9^ Mean platelet volume (MPV) has been regarded as a surrogate marker of platelet activation.^10^ MPV was a useful prognostic indicator for critical patients.^11^, ^12^, ^13^ Besides, some studies have shown a negative correlation between MPV and platelet count (PC) in severe patients.^14^, ^15^ It is reported that the combination of MPV and PC could be more clinically significant than MPV or PC alone.^14^, ^16^ As far as we know, no published article investigated the relationship between MPV/platelet count ratio (MPR) and the prognosis of COVID‐19. Our research aims to assess whether MPR is a useful predictor for the progression of COVID‐19.

## 2 MATERIALS AND METHODS

### 2.1 Study participants

From January 21, 2020, to February 14, 2020, patients who suffered from COVID‐19 were recruited from two hospitals in southern China. Patients who were younger than 18 or suffered from liver diseases were excluded from this study. This retrospective cohort study was approved by the Ethics Committee. Written informed consent was waived.

### 2.2 Definitions

COVID‐19 was diagnosed and classified based on the guidance from the World Health Organization (WHO).^17^ SP of COVID‐19 was defined as fever or suspected respiratory infection, plus one of the following: (a) respiratory rate >30 breaths/min, (b) severe respiratory distress, and (c) SpO2 ≤ 93% on room air.

### 2.3 Data collection

Epidemiological, clinical, laboratory, radiological data were collected from case report forms and electronic medical records. The information recorded included demographic data, comorbid conditions, exposure history, signs, laboratory data, chest computed tomographic (CT) scans, and treatments. Laboratory findings, Chest CT results, and treatment measures during the hospital stay were monitored. Baseline laboratory data were collected on admission to the hospital. Samples of peripheral blood were collected into tubes with ethylenediamine tetraacetic acid. Mean platelet volume and platelet distribution width were determined using an automated blood cell analyzer (XN‐2000, Sysmex) within 30 minutes of sample collection. MPR was defined as mean platelet volume (fL)/platelet count (^10^9^/L) * 100%. Health‐care providers or responsible doctors were contacted for clarification if data were missing or not clear from records.

### 2.4 Follow‐up

After admission, patients were re‐examined for laboratory data and CT if necessary. Clinical outcomes were monitored up to February 19, 2020, the final date of follow‐up. Patients were censored if they were transferred, discharged, or still hospitalized without SP until the final date of follow‐up. The primary outcome of this study was the SP developed.

### 2.5 Statistical analysis

Patients were divided into low and high MPR group according to the optimal value of baseline MPR for predicting SP developed. Baseline data were expressed as mean ± standard deviation or median (interquartile range) for continuous data, and number (%) for categorical data. Differences between two groups were compared using Student's *t* test or Mann‐Whitney *U* test for continuous data, and chi‐square test, Fisher's exact test, or Kruskal‐Wallis test for categorical data. The receiver operating characteristic (ROC) curve was performed to assess the predictive ability of baseline MPR for the incidence of SP and to acquire MPR cutoff values to maximize sensitivity and specificity. Spearman's correlation coefficients were used to assess the relationships between MPR and other clinical variables. The generalized estimating equations were conducted to compare data from repeated measures. Kaplan‐Meier method was used to estimate the incidence of SP, and differences between two groups were compared by the log‐rank test. The relationships of SP developed and MPR were determined by univariate and backward stepwise multivariate Cox regression analysis. Age, sex, center, and variables with *P* < .1 in univariate analysis were entered into the multivariate model. In Cox models, time at risk was from study entry till SP developed, discharge, transfer, or the final date of follow‐up. To reduce the influence of unbalanced distribution of confounding factors, the weighted Cox regression analysis was used to adjust confounders through the inverse probability of treatment weighting (IPTW). The propensity score was calculated in each case by a logistic regression model including factors in the Cox model. Remaining missing data were filled using the missForest R package. All statistical analyses were performed using SPSS (Statistical Package for the Social Sciences) version 25.0 software (SPSS Inc) and R software (version R‐3.5.5, www.r-project.org). *P* < .05 were considered statistically significant.

## 3 RESULTS

### 3.1 Baseline characteristics

**FIGURE 1:**
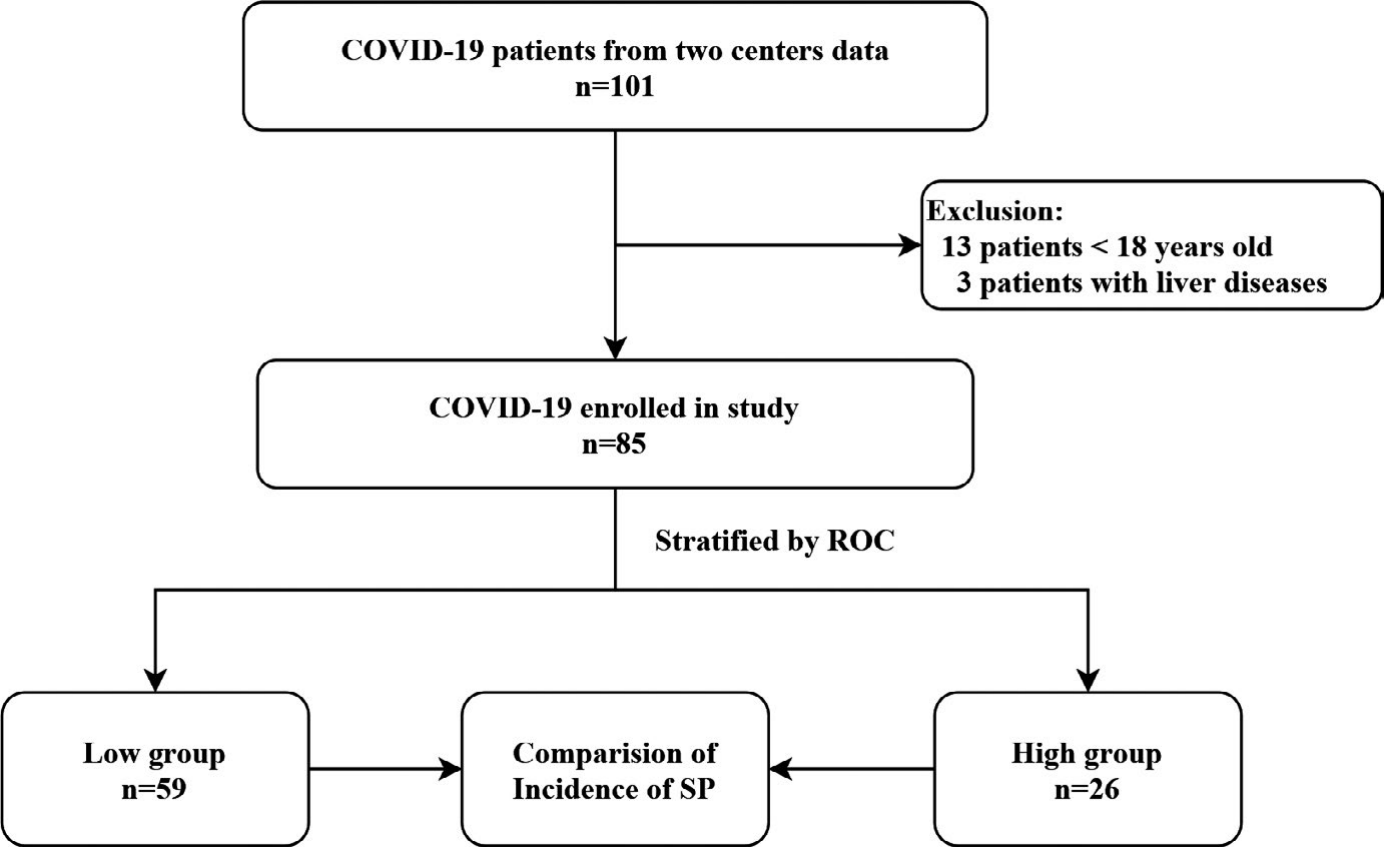
Study flow diagram. By February 14, 2020, 101 patients with confirmed COVID‐19 had been admitted to two centers. 85 patients were ultimately enrolled in this study. Note: Low group, MPR ≤ 7.44 group; high group, MPR > 7.44 group. ROC, Receiver operating characteristic curve analysis; SP, severe pneumonia

By February 14, 2020, 101 patients with confirmed COVID‐19 had been admitted, of whom 16 (12.9%) were considered ineligible due to age < 18 or liver diseases. 85 patients were ultimately enrolled in this study (Figure 1). The demographics and characteristics data of two groups, categorized based on the optimal cutoff value of baseline MPR for predicting SP developed, were presented in Table 1. The median age was 43.0 (34.5, 61.5), and 44 (51.8%) were male. The median days from symptom onset to admission were 3.0 (2.0, 5.0) days. No patients had been exposed to the Wuhan seafood market but 28 (32.9%) had made short‐term trips to Wuhan city before illness onset. Patients in the high MPR group showed an increased prevalence of hypertension (26.9% vs 6.8%, *P* = .030), but no significant difference was observed in other comorbid conditions between two groups. Among patients with SP developed, the median days from admission to SP were 6.0 (5.0, 11.5) days. Among those discharged with mild illness, the median duration of hospital stay was 14.0 (13.0, 19.0) days. All patients were diagnosed as mild illness or pneumonia of COVID‐19 on admission. During follow‐up, 17 (20.0%) patients were developed to SP, with a significantly higher incidence in the high MPR group than the low MPR group (42.3% vs 10.2%, *P* = .001). At the end of February 19, 2020, 64 (75.3%) patients were still hospitalized, 20 (23.5%) patients were discharged, and one patient (1.2%) died during hospitalization.

**TABLE 1:** Baseline characteristics between two MPR levels

	Total (n = 85)	MPR ≤ 7.44 (n = 59)	MPR > 7.44 (n = 26)	*P*‐value
No. of C1/C2	73/12	51/8	22/4	NA
MPR (%)	6.4 ± 2.1	5.2 ± 1.0	9.0 ± 1.6	NA
Demographics				
Age (y)	43.0 (34.5, 61.5)	41.0 (34.0, 58.0)	51.0 (35.5, 68.3)	0.064
Sex (male)	44 (51.8%)	25 (42.4%)	19 (73.1%)	0.009
Comorbid conditions				
Hypertension	11 (12.9%)	4 (6.8%)	7 (26.9%)	0.030
Diabetes	2 (2.4%)	1 (1.7%)	1 (3.8%)	0.521
Cardiovascular disease	2 (2.4%)	0 (0.0%)	2 (7.7%)	0.091
Cerebrovascular disease	1 (1.2%)	0 (0.0%)	1 (3.8%)	0.306
COPD	0 (0.0%)	0 (0.0%)	0 (0.0%)	NA
Renal diseases	1 (1.2%)	1 (1.7%)	0 (0.0%)	1.000
Smoke	4 (4.7%)	3 (5.1%)	1 (3.8%)	1.000
Exposure history in Wuhan <2 wk	28 (32.9%)	20 (33.9%)	8 (30.8%)	0.777
From symptom onset to admission (d)	3.0 (2.0, 5.0)	3.0 (2.0, 5.0)	3.0 (2.0, 4.3)	0.836
Temperature (°C)	37.2 (36.7, 37.7)	37.2 (36.7, 37.6)	37.2 (36.7, 37.9)	0.560
SBP (mmHg)	126.9 ± 15.3	124.6 ± 14.6	132.2 ± 15.7	0.035
DBP (mmHg)	83.7 ± 9.6	82.1 ± 8.9	87.3 ± 10.2	0.019
Treatment				
Antibiotic therapy	78 (91.8%)	54 (91.5%)	24 (92.3%)	1.000
Use of corticosteroid	38 (44.7%)	24 (40.7%)	14 (53.8%)	0.261
Arbidol + lopinavir/ritonavir	70 (82.4%)	48 (81.4%)	22 (84.6%)	1.000
Oseltamivir/ribavirin	31 (36.5%)	17 (28.8%)	14 (53.8%)	0.027
Interferon alpha	62 (72.9%)	42 (71.2%)	20 (76.9%)	0.583
Gamma globulin	14 (16.5%)	6 (10.2%)	8 (30.8%)	0.027
Thymosin	54 (63.5%)	35 (59.3%)	19 (73.1%)	0.225
Chloroquine	25 (29.4%)	18 (30.5%)	7 (26.9%)	0.738
Oxygen therapy	76 (89.4%)	52 (88.1%)	24 (92.3%)	0.715
Chest CT findings				
Normal	8 (9.4%)	8 (13.6%)	0 (0.0%)	0.013
Unilateral, single pneumonia	8 (9.4%)	7 (11.9%)	1 (3.8%)	
Unilateral, multiple pneumonia	5 (5.9%)	4 (6.8%)	1 (3.8%)	
Bilateral, multiple pneumonia	64 (75.3%)	40 (67.8%)	24 (92.3%)	
Blood laboratory findings				
WBC (10^9/L)	5.0 ± 1.7	5.3 ± 1.7	4.4 ± 1.4	0.021
Neutrophil (10^9/L)	3.4 ± 1.5	3.6 ± 1.6	3.0 ± 1.2	0.064
Lymphocyte (10^9/L)	1.1 (0.8, 1.5)	1.1 (0.9, 1.5)	0.9 (0.7, 1.4)	0.031
Monocyte (10^9/L)	0.4 ± 0.1	0.4 ± 0.1	0.4 ± 0.2	0.702
Hb (g/L)	139.4 ± 17.1	137.3 ± 18.5	144.1 ± 12.6	0.090
HCT (%)	41.1 ± 4.0	40.6 ± 4.2	42.2 ± 3.5	0.108
Platelet (10^9/L)	193.2 ± 52.2	218.3 ± 40.4	136.4 ± 22.8	<0.001
MPV (fL)	11.3 ± 1.0	11.0 ± 0.9	12.0 ± 1.1	<0.001
PDW (%)	13.2 (12.0, 14.8)	12.6 (11.8, 13.9)	14.6 (13.1, 16.8)	0.004
AST (U/L)	25.0 (18.0, 33.0)	24.0 (18.0, 33.0)	26.4 (22.5, 33.5)	0.244
ALT (U/L)	18.9 (12.8, 27.6)	18.5 (12.5, 26.4)	21.4 (13.0, 29.3)	0.477
Albumin (g/L)	40.5 ± 3.6	41.2 ± 3.2	39.0 ± 4.1	0.010
Globulin (g/L)	27.5 (25.3, 29.4)	27.5 (25.2, 29.9)	27.1 (25.1, 28.6)	0.429
BUN (mmol/L)	4.0 (3.2, 5.0)	3.8 (3.2, 4.5)	4.5 (3.3, 5.9)	0.072
Creatinine (μmol/L)	68.3 (55.0, 77.9)	63.0 (55.0, 75.0)	74.5 (65.8, 84.0)	0.030
eGFR (mL/(min·1.73 m^2^))	109.1 (97.8, 119.6)	110.1 (100.0, 124.8)	106.2 (95.2, 115.5)	0.285
PH	7.4 ± 0.0	7.4 ± 0.0	7.4 ± 0.0	0.258
PO2 (mmHg)	111.0 (85.8, 152.0)	111.0 (85.8, 145.0)	105.4 (81.7, 165.3)	0.812
PCO2 (mmHg)	39.1 ± 3.9	39.3 ± 3.9	38.7 ± 3.9	0.546
BE (mmol/L)	0.6 (−0.4, 2.5)	0.3 (−0.5, 2.5)	1.2 (0.1, 2.7)	0.241
Hs‐CRP (mg/L)				
<0.5	14 (16.5%)	14 (23.7%)	0 (0.0%)	0.006
0.5‐5.0	25 (29.4%)	18 (30.5%)	7 (26.9%)	
>5.0	46 (54.1%)	27 (45.8%)	19 (73.1%)	
Prognosis				
Hospitalization	64 (75.3%)	44 (74.6%)	20 (76.9%)	0.261
Discharge	20 (23.5%)	15 (25.4%)	5 (19.2%)	
Death	1 (1.2%)	0 (0.0%)	1 (3.8%)	
From admission to SP developed^a^ (d)	6.0 (5.0, 11.5)	8.0 (4.0, 10.8)	6.0 (5.0, 14.0)	0.840
Duration of hospital stay^b^ (d)	14.0 (13.0, 19.0)	14.0 (13.0, 18.3)	19.0 (14.0, 20.5)	0.189
SP developed	17 (20.0%)	6 (10.2%)	11 (42.3%)	0.001

Note

Data are presented as mean ± standard deviation, median (interquartile range) or n (%).

Abbreviations: ALT, alanine aminotransferase; AST, aspartate aminotransferase; BE, base excess of extracellular fluid; BUN, blood urea nitrogen; C1, center 1; C2, center 2; COPD, chronic obstructive pulmonary disease; DBP, diastolic blood pressure; eGFR, estimated glomerular filtration rate; Hb, hemoglobin; HCT, hematocrit; Hs‐CRP, high sensitive C‐reactive protein; MPR, mean platelet volume to platelet count ratio; MPV, mean platelet volume; PDW, platelet distribution width; SBP, systolic blood pressure; SP, severe pneumonia; WBC, white blood cell.

^a^ Only for patients with SP developed.

^b^ Only for patients discharged with mild illness.

Abnormalities on chest CT were detected among 77 (90.6%) patients on admission. 8 (9.4%) had unilateral, single pneumonia, 5 (5.9%) had unilateral, multiple pneumonia, and 64 (75.3%) had bilateral, multiple pneumonia. Notably, 8 (9.4%) had no typical chest CT findings, and none of them was in the high MPR group. Patients from the high MPR group had a significantly greater proportion of bilateral, multiple pneumonia on admission (92.3% vs 67.8%, *P* = .009).

Most patients received antibiotic therapy (78, 91.8%) and antiviral therapy (Arbidol + lopinavir/ritonavir (70, 82.4%); Oseltamivir/ribavirin (31, 36.5%); Interferon alpha (62, 72.9%)), while high MPR group showed more proportion of administration using oseltamivir/ribavirin (53.8% vs 28.8%, *P* = .027). 38 (44.7%) patients were given corticosteroid. Gamma globulin was used in 14 (16.5%) patients, and thymosin was applied in 54 (63.5%) patients. In addition, 25 (29.4%) patients were given chloroquine, and 76 (89.4%) patients were supplied with oxygen.

There were significant differences in laboratory findings on admission between two MPR levels. Patients in high MPR group showed lower white blood cell, lymphocyte, platelet, and albumin (*P* = .021, *P* = 0 0.031, *P* < .001, and *P* = .010, respectively), and higher MPV, PDW, creatinine, and hs‐CRP (*P* < .001, *P* = .004, *P* = .030, and *P* = .006, respectively).

### 3.2 Performance of baseline MPR as a predictor of SP developed by ROC curve analysis

**FIGURE 2:**
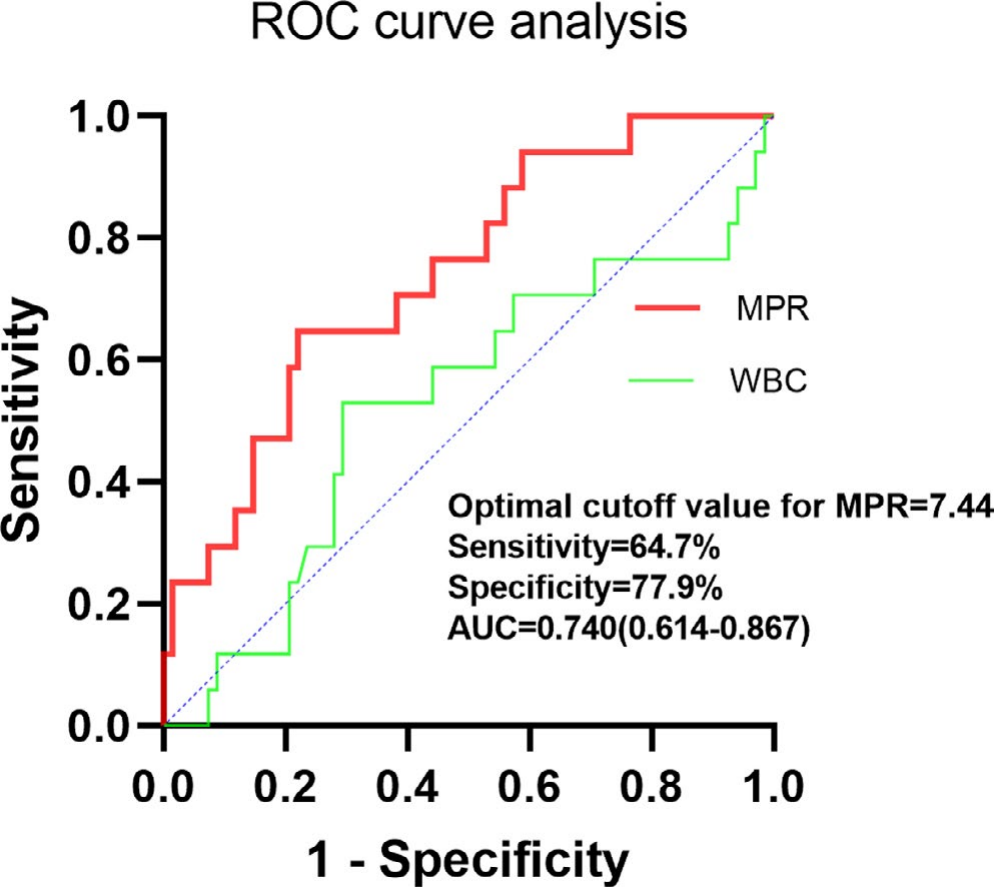
ROC curves of MPR and WBC for predicting SP. MPR had a modest power for predicting SP as suggested by AUC of 0.740 (95% CI, 0.614‐0.867, *P* = .002). The optimal cutoff value of MPR for predicting SP was 7.44% (Sensitivity = 64.7%, Specificity = 77.9%)

The area under curve (AUC) of the baseline MPR and WBC for the incidence of SP were 0.740 (95% CI, 0.614‐0.867, *P* = .002) and 0.527 (95% CI, 0.364‐0.690, *P* = .729). The cutoff values of MPR for predicting SP developed were 7.44, with a sensitivity of 64.7% and specificity of 77.9% (Figure 2).

### 3.3 Correlations between baseline MPR and clinical variables

The correlations between baseline MPR and clinical variables were presented in Table 2 using the Spearman correlation coefficient. MPR was positively correlated with age (*r* = .261, *P* = .016), hypertension (*r* = .239, *P* = .028), cardiovascular diseases (CVD) (*r* = .218, *P* = .045), Hb (*r* = .261, *P* = .016), AST (*r* = .241, *P* = .026), BUN (*r* = .215, *P* = .048), creatinine (*r* = .254, *P* = .019), hs‐CRP (*r* = .391, *P* < .001), and involvement on chest CT (*r* = .238, *P* = .028). Moreover, MPR was negatively correlated with female (*r* = −.316, *P* = .003), lymphocyte (*r* = −.260, *P* = .016), and albumin (*r* = −.331, *P* = .002).

**TABLE 2:** Correlations between baseline MPR and clinical parameters

Variables	*r*	*P*‐value
Age	.261	.016
Female	−.316	.003
Hypertension	.239	.028
Cardiovascular disease	.218	.045
WBC	−.212	.052
Neutrophil	−.131	.234
Lymphocyte	−.260	.016
Hb	.261	.016
AST	.241	.026
ALT	.160	.144
Albumin	−.331	.002
BUN	.215	.048
Creatinine	.254	.019
Hs‐CRP	.391	<.001
Involvement (CT findings)	.238	.028

Abbreviations: ALT, alanine aminotransferase; AST, aspartate aminotransferase; BUN, blood urea nitrogen; Hb, hemoglobin; Hs‐CRP, high sensitive C‐reactive protein; MPR, mean platelet volume to platelet count ratio; WBC, white blood cell.

### 3.4 Dynamic profile of MPR in patients with COVID‐19

**FIGURE 3:**
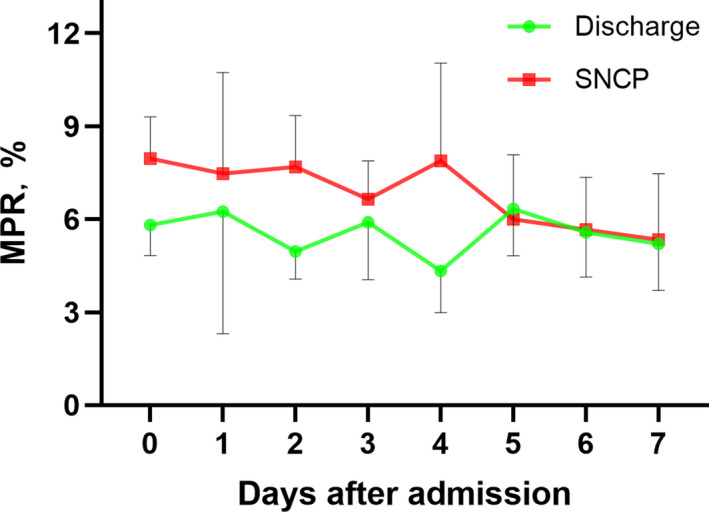
Dynamic profile of MPR in 36 patients with COVID‐19 during the first week after admission. Compared with patients discharged without SP, patients with SP developed showed a higher MPR level at baseline (*P* = .005 vs Discharge), day 1 (*P* = .015 vs Discharge), day 2 (*P* = .009 vs Discharge), and day 3 (*P* = .032 vs Discharge) after admission. The MPR values of each day from patients with SP or discharged were showed as the median and upper or lower limit of 95% confidence interval

To compare the dynamic changes of MPR between mild and severe patients in the early stage, the MPR data were tracked from day 0 to day 7 after admission at one‐day intervals (Figure 3). At the end of February 19, 2020, data from 36 patients (19 discharged without SP and 17 patients with SP developed) were analyzed using generalized estimating equations. Compared with patients discharged without SP, patients with SP developed showed a higher MPR level at baseline (*P* = .005 vs Discharge), day 1 (*P* = .015 vs Discharge), day 2 (*P* = .009 vs Discharge), and day 3 (*P* = .032 vs Discharge) after admission. Besides, the MPR level at baseline did not change during the first week in the discharge group, but decreased significantly in the SP group on day 5 (*P* = .014 vs baseline), day 6 (*P* < .001 vs baseline), and day 7 (*P* = .001 vs baseline) after admission.

### 3.5 MPR associated with SP developed

**FIGURE 4:**
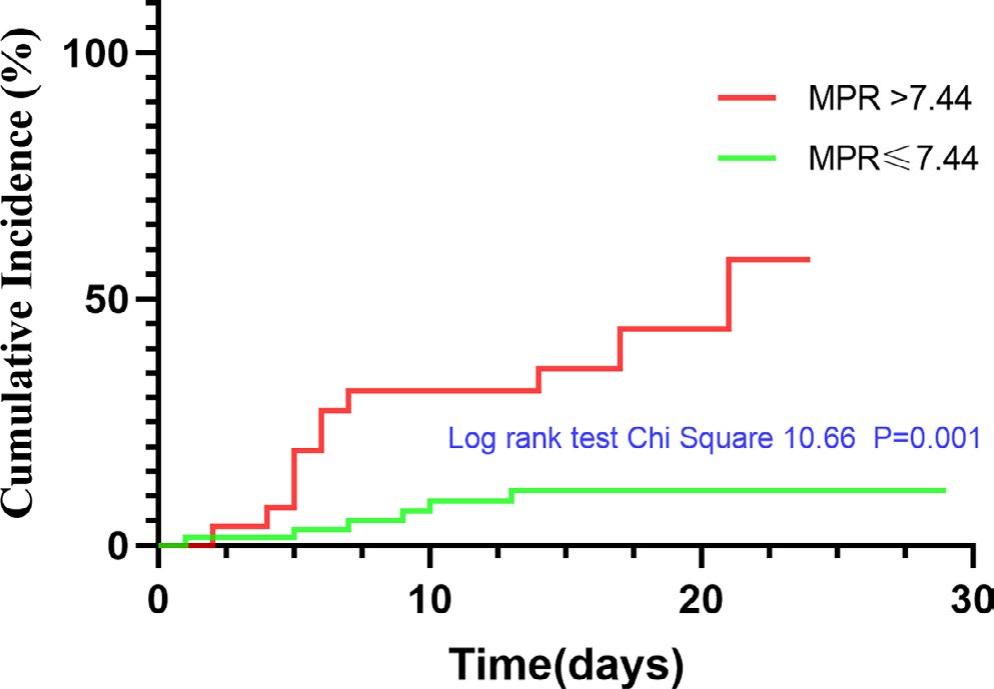
Cumulative incidence curves for SP developed by the optimal cutoff value of MPR. The curves were constructed using Kaplan‐Meier method and compared using the Mantel‐Cox log‐rank test. Compared to patients in the low MPR group (≤7.44), patients in high MPR group (MPR > 7.44) showed a significant elevated incidence of SP of COVID‐19 (Log‐rank test = 10.66, *P* = .001)

Kaplan‐Meier curves for MPR level based on the optimal cutoff value were shown in Figure 4. Patients in the high MPR group had an obviously higher incidence of SP than those in the low MPR group during the course of their hospitalization (log‐rank test *χ* ^2^ = 10.66, *P* = .001).

The relationship of MPR with SP incidence with defined models (with low MPR group as the reference group) was shown in Table 3. Despite the adjustment, MPR > 7.44 was associated with a higher incidence of SP. In model 3, which was a maximally adjusted model including center, age, sex, hypertension, temperature, use of corticosteroid, gamma globulin, AST, ALT, albumin, BUN, eGFR, PCO2, PO2, adjusted HRs for developing to SP were 5.841 (95% CI, 1.566‐21.791, *P* = .009). Moreover, implementation of the balance between two groups by IPTW also suggested that MPR as a significant factor associated with the incidence of SP (IPTW‐adjusted HRs, 8.337, 95% CI, 4.045‐17.182, *P* < .001).

**TABLE 3:** Relationship between MPR with SP

	MPR > 7.44 group	
	HR (95% CI)	*P*
Unadjusted	4.517 (1.665‐12.250)	.003
Model 1	2.653 (0.930‐7.566)	.068
Model 2	3.742 (1.278‐10.960)	.016
Model 3	5.841 (1.566‐21.791)	.009
IPTW	8.337 (4.045‐17.182)	<.001

Note

The reference group is MPR ≤ 7.44 group. Covariates included age, sex, center, and variables with *P* < .1 in univariate Cox analysis.

Model 1: Center, Age, Sex.

Model 2: Model 1 covariates, hypertension, temperature, and treatment (use of corticosteroid and gamma globulin).

Model 3: Model 2 covariates and laboratory data (AST, ALT, Albumin, BUN, eGFR, PCO2, PO2).

Abbreviations: ALT, alanine aminotransferase; AST, aspartate aminotransferase; BUN, blood urea nitrogen; eGFR, estimated glomerular filtration rate; IPTW, inverse probability of treatment weighting; MPR, mean platelet volume to platelet count ratio; SP, severe pneumonia.

## 4 DISCUSSION

In this study, we found that even after adjusting for multiple confounding factors, the increased baseline MPR was apparently associated with the progression of COVID‐19. Moreover, patients with SP developed tended to have higher MPR levels than mild patients in the early stage after admission. Recent studies indicated that monocyte‐to‐lymphocyte ratio could be used to differentiate COVID‐19 patients from healthy subjects and neutrophil to lymphocyte ratio might be a reliable marker to assess the severity of COVID‐19.^18^ These suggested that the dynamic change of hemocytes was of great clinical significance and became a research hotspot. Up till now, no study investigated the prognostic value of MPR for COVID‐19. Thus, we aim to investigate the association between MPR and COVID‐19.

COVID‐19 particles spread through the respiratory mucosa, leading to viral pneumonia.^4^, ^5^ The differences in CT results and high sensitive C‐reactive protein (hs‐CRP) between two MPR groups were statistically significant. CT showed that 26 patients had a pulmonary infection in the high MPR group, including one patient (3.8%) with unilateral pneumonia, one patient (3.8%) with unilateral multiple pneumonia, and 24 patients (92.3%) with bilateral multiple pneumonia. Compared to the high MPR group, the low MPR group showed less proportion of patients with pulmonary infection. Our laboratory tests showed that 19 patients (73.1%) had an inflammation index of hs‐CRP > 5.0 in high MPR group, while only 27 patients (45.8%) in the low MPR group. Spearman's analysis also revealed a significant positive correlation between MPR and CRP, as well as MPR and CT involvement. Our results suggested that the high MPR was more seriously related to pulmonary infection and inflammation in patients with COVID‐19.

Platelets are essential for inflammation, hemostasis, and immune regulation.^19^, ^20^ Platelet activation plays a crucial role in viral pneumonia.^21^ In viral pneumonia, platelet activation may cause lung injury by stimulating the detrimental inflammatory response of respiratory tract.^22^ Activated platelets not only release inflammatory mediators but also expose surface molecules (including E‐selectin and P‐selectin), which affect the interaction between platelets and other cells, causing systemic inflammation and immune response.^9^ The decrease in PC is associated with poor prognosis and increased mortality in hospitalized patients.^23^, ^24^ In contrast, PC elevation is bound up with a good prognosis.^25^ Besides, for pneumonia patients, thrombocytopenia is a sign of adverse prognosis in hospitalized pneumonia patients and intensive care unit (ICU) patients hospitalized with severe community‐acquired pneumonia (CAP).^26^, ^27^ Previous studies have revealed that the increase of MPV is relevant to the adverse prognoses of patients, such as CVD, chronic inflammatory disease, severe pneumonia, septic shock, and other diseases.^27^, ^28^, ^29^, ^30^, ^31^ In patients with severe pneumonia, the increase of MPV after admission may be able to forecast the mortality.^32^, ^33^

MPV is negatively correlated with PC in severe patients.^15^ MPR, which seems to be a better indicator of platelet function, has been proposed.^12^, ^14^, ^20^, ^30^, ^31^, ^34^ In many diseases, the increase of MPR is obviously associated with adverse prognoses, such as pneumonia after ischemic stroke, sepsis, critical disease, CVD, febrile epilepsy in children, and malignant tumor.^15^, ^20^, ^34^, ^35^ Ranias et al have shown that if pneumonia occurs in patients with ischemic stroke, an increase in the MPV/PC ratio could predict 30 days mortality.^35^ Our study suggested that the increase of MPR was also a predictor of poor prognosis in patients with pneumonia caused by COVID‐19.

The pathophysiological mechanism of MPR in predicting the adverse prognosis of patients with COVID‐19 is not clear, but it may be related to the following mechanisms. Firstly, under the condition of inflammation, platelet production will increase owing to the increased synthesis of thrombopoietin mediated by multifarious cytokines.^22^, ^36^, ^37^ Secondly, MPV reflects the metabolism and proliferation of megakaryocytes and platelet production in bone marrow.^9^, ^13^, ^21^ In the beginning, when infection occurs, the release of many inflammatory cytokines (such as interleukin‐1 (IL‐1), IL‐3 and IL‐6, and tumor necrosis factor‐α (TNF‐α)) increases, leading to the increase of thrombopoietin and the expression of young platelets in the blood stream,^9^, ^32^ which causes the increase of MPV. Besides, after stress‐induced platelet destruction, the decrease of PC further stimulates megakaryocyte to produce a large number of platelets, which also leads to an increase of MPV.^38^ Thirdly, adverse prognosis in patients with decreased PC and elevated MPV may be associated with increased risk of oxidative stress, thrombosis, and apoptosis in activated platelets.^26^, ^27^

Our research has certain limitations. Firstly, this was a single ethnic group study. Whether these findings can be applied to other ethnic groups remains to be explored. Secondly, our study sample included patients from two designated hospitals in Guangdong Province. There were no data from Hubei Province, the most serious epidemic area in China. Thirdly, owing to the retrospective nature, it is hard to define an absolute range of MPR to differentiate mild patients from severe illness. Finally, the relatively small sample size is a chief limitation of this study. Due to the small sample, it should be cautious to make a conclusion. But as we observed a significant association between MPR and the incidence of SP after using several methods to reduce probable bias and confounders, we suggested that clinicians should note the possibility of a higher risk of poor outcomes in this situation. Further large‐scale prospective studies are needed to confirm the association between MPR and COVID‐19.

In conclusion, our study suggested that MPR is a useful indicator to help predict whether COVID‐19 patients will progress to severe pneumonia. The early application of MPR is conducive to the hierarchical management of patients' risks and alleviates the shortage of medical resources.

## CONFLICT OF INTEREST

The authors declare that they have no financial conflicts of interest. Human and animal rights All procedures performed in studies involving human participants were in accordance with the ethical standards of the institutional and/or national research committee at which the studies were conducted (IRB approval number 2020‐hg‐ks‐04), and with the 1964 Helsinki Declaration and its later amendments or comparable ethical standards.
